# Incidence and molecular characteristics of deficient mismatch repair conditions across nine different tumors and identification of germline variants involved in Lynch-like syndrome

**DOI:** 10.1007/s10147-024-02518-y

**Published:** 2024-04-14

**Authors:** Tetsuya Ito, Tatsuro Yamaguchi, Kensuke Kumamoto, Okihide Suzuki, Noriyasu Chika, Satoru Kawakami, Tomonori Nagai, Tsukasa Igawa, Kenji Fujiyoshi, Yoshito Akagi, Tomio Arai, Kiwamu Akagi, Hidetaka Eguchi, Yasushi Okazaki, Hideyuki Ishida

**Affiliations:** 1 Department of Digestive Tract and General Surgery, Saitama Medical Center, Saitama Medical University, 1981 Kamoda, Kawagoe, Saitama 350-8550 Japan; 2 Department of Surgery, Tokyo Metropolitan Cancer and Infectious Diseases Center, Komagome Hospital, Tokyo, Japan; 3 Department of Gastroenterological Surgery, Kagawa University, Kagawa, Japan; 4 Department of Clinical Genetics, Saitama Medical Center, Saitama Medical University, Saitama, Japan; 5 Department of Urology, Saitama Medical Center, Saitama Medical University, Saitama, Japan; 6 Department of Obstetrics and Gynecology, Saitama Medical Center, Saitama Medical University, Saitama, Japan; 7 Department of Urology, Kurume University School of Medicine, Kurume, Japan; 8 Department of Surgery, Kurume University, Kurume, Japan; 9 Department of Pathology, Tokyo Metropolitan Geriatric Institute for Geriatrics and Gerontology, Tokyo, Japan; 10 Division of Molecular Diagnosis and Cancer Prevention, Saitama Cancer Center, Saitama, Japan; 11 Diagnostics and Therapeutics of Intractable Diseases and Intractable Disease Research Center, Juntendo University Graduate School of Medicine, Tokyo, Japan

**Keywords:** Deficient DNA mismatch repair (dMMR), dMMR tumors, Lynch-like syndrome

## Abstract

**Background:**

Based on molecular characteristics, deficient DNA mismatch repair (dMMR) solid tumors are largely divided into three categories: somatically *MLH1*-hypermethylated tumors, Lynch syndrome (LS)-associated tumors, and Lynch-like syndrome (LLS)-associated tumors. The incidence of each of these conditions and the corresponding pathogenic genes related to LLS remain elusive.

**Methods:**

We identified dMMR tumors in 3609 tumors from 9 different solid organs, including colorectal cancer, gastric cancer, small-bowel cancer, endometrial cancer, ovarian cancer, upper urinary tract cancer, urinary bladder cancer, prostate cancer, and sebaceous tumor, and comprehensively summarized the characterization of dMMR tumors. Characterization of dMMR tumors were performed as loss of at least one of MMR proteins (MLH1, MSH2, MSH6, and PMS2), by immunohistochemistry, followed by *MLH1* promotor methylation analysis and genetic testing for MMR genes where appropriate. Somatic variant analysis of MMR genes and whole exome sequencing (WES) were performed in patients with LLS.

**Results:**

In total, the incidence of dMMR tumors was 5.9% (24/3609). The incidence of dMMR tumors and the proportion of the three categorized dMMR tumors varied considerably with different tumor types. One to three likely pathogenic/pathogenic somatic MMR gene variants were detected in 15 out of the 16 available LLS tumors. One patient each from 12 patients who gave consent to WES demonstrated non-*MMR* germline variants affect function (*POLQ* or *BRCA1*).

**Conclusions:**

Our data regarding the LS to LLS ratio would be useful for genetic counseling in patients who are suspected to have LS, though the genetic backgrounds for the pathogenesis of LLS need further investigation.

**Supplementary Information:**

The online version contains supplementary material available at 10.1007/s10147-024-02518-y.

## Introduction

Recently, utilization of immune checkpoint inhibitors in the treatment of DNA mismatch repair deficient (dMMR) solid tumors has gained significant attention [1, 2]. Currently, two major methods are used to identify dMMR in the tumors, molecular testing of microsatellite instability (MSI) and immunohistochemistry (IHC) of MMR proteins (MLH1, MSH2, MSH6, and PMS2). dMMR is usually defined as high-frequency MSI (MSI-H) or loss of expression (MMR-D) of at least one MMR protein. MSI-H and MMR-D yield high concordant results (95–99%) in colorectal cancer (CRC) and endometrial cancer (EC) [3, 4].

Based on molecular characteristics, MMR-D and/or MSI-H solid tumors are divided into three categories: 1. somatically *MLH1*-hypermethylated tumors, Lynch syndrome (LS)-associated tumors, and Lynch-like syndrome (LLS)-associated tumors. *MLH1*-hypermethylated tumor is the most common type, it is non-heritable and caused by aberrant hypermethylation of the *MLH1* promoter region, leading transcriptional inactivation [5, 6]. LS is an autosomal dominant inherited disorder, mainly caused by a germline pathogenic variant of MMR genes, though 3′ deletion of *EPCAM* located upstream of *MSH2* is also known to be another cause of LS [7]. Biallelic inactivation of MMR genes by germline and somatic variants leads to a high frequency of replication errors in microsatellite regions and repeated sequences in the coding regions of various, growth-related target genes [8], resulting in the development of dMMR tumors in various organs. LLS has recently been proposed as a third dMMR tumor type that do not harbor germline MMR gene variants or *MLH1* hypermethylation [9, 10]. Somatic MMR gene variants frequently occur in LLS [11, 12]. However, there have been a limited number of reports [13] regarding MMR unrelated germline pathogenic variants in LLS tumorigenesis.

Recently, several large-scale investigations have reported the incidence of MSI-H among various solid tumors [14, 15]. However, the proportion of sporadic dMMR, LS-associated tumors (LS-AT), and LLS-associated tumors (LLS-AT), along with the proportion of dMMR-associated MMR genes, remains unknown. In addition, germline pathogenic variants unrelated to MMR have rarely been investigated. Using MMR-IHC, we previously performed universal tumor screening for identification of LS, and reported the proportion of at least three categorized dMMR tumors in nine different tumors, including CRC, gastric cancer (GC), small-bowel cancer (SBC), EC, ovarian cancer (OC), upper urinary tract cancer (UUTC), urinary bladder cancer (UBC), prostate cancer (PC), and sebaceous tumor (ST) [12, 16–23]. In the present study, we comprehensively analyzed data from previous publications. Furthermore, using whole exome sequencing, we investigated the presence of non-*MMR* germline pathogenic variants in LLS tumorigenesis.

## Patients and methods

### Ethical consideration

The current study and the associated studies (previously published) [12, 16–23] have been approved by the local ethics committee of the Saitama Medical Center (No. 924, No. 925, and No. 926) and the Saitama Medical University (No. 592 and No. 747). Informed consent was obtained from all patients before *MMR* genetic testing and whole exome sequencing. Consent was obtained from family members of deceased patients.

### Patients

In this study, we analyzed a total of 3609 tumor specimens, resected from nine different solid organs: CRC (n = 1234), GC (n = 513), SBC (n = 30), EC (n = 395), OC (n = 305), UUTC (n = 164), UBC (n = 618), PC (n = 337), and ST (n = 13) (supplementary Table 1). All tumor specimens were resected and stored at Saitama Medical Center of Saitama Medical University.

### IHC for MMR proteins

IHC was performed to examine the expression of four MMR proteins (MLH1, MSH2, MSH6, and PMS2). Tumor sections (4 μm) were fixed with formalin and embedded in paraffin (FFPE). Then, the sections were stained using a Staining Automat (BOND III, Leica Biosystems Melbourne Pvt. Ltd, Melbourne, Australia), according to the manufacturer’s protocol. The antibodies used for detecting MMR proteins were described previously [12, 16–23].

The normal expression pattern for MMR proteins is nuclear. Complete loss of nuclear expression in tumor cells with the presence of nuclear expression in non-neoplastic cells, such as normal epithelial cells, lymphocytes, or stromal cells was considered to represent an abnormal pattern (MMR-D).

### Classification of dMMR tumors

#### MLH1-hypermethylated sporadic tumors

Methylation analysis of the *MLH1* promoter region was performed in the FFPE tumor specimens using real-time PCR-based, MethyLight, or bisulfite-Sanger sequencing; as previously described [12, 16–23]. Based on the Sanger sequencing results, samples were classified as hypermethylated, heterozygously methylated, or unmethylated [12]. In the present study, “heterogeneously methylated” tumors were classified as “unmethylated”. MethyLight analysis was used to determine the percentage of methylated reference (PMR). Based on validated data, a positive PMR cut-off of 10% was used. Therefore, samples were considered positive if the PMR was > 10%.

During CRC analysis [12], *MLH1*-methylation analysis was omitted in the tumor samples exhibiting *BRAF*V600E. However, subsequent MethyLight analysis confirmed that these tumors were methylation positive.

As a major principle, we did not perform LS genetic testing for patients with *MLH1*-methylated tumors. However, two patients with *MLH1*-methylated tumors underwent genetic testing: Patient 1, a 50-year-old man with CRC exhibiting loss of function of the MLH1/PMS2 protein dimer, with heterogeneously methylated molecular characteristics. A germline *MLH1* pathogenic variant was confirmed, leading to an LS diagnosis. Patient 2, a 70-year-old man with SC exhibiting MLH1/PMS2 and *MLH1*-methylated results. Due to the lack of sufficient dMMR data and aberrant *MLH1* methylation in the field of ST, genetic testing was performed to confirm the patient’s LS status [23].

#### Lynch syndrome-associated tumors

Following genetic counseling and informed consent, we conducted genetic testing: Sanger sequencing and multi-gene panel sequencing with/without RNA-sequencing using DNA from blood leukocytes where appropriate. If only FFPE tissue specimens were available, Sanger sequencing was performed to determine MMR genes, according to the IHC patterns of the MMR proteins. A multiplex ligation-dependent probe amplification (MLPA) method was used to analyze the copy number variation of the *MLH1*, *MSH2*, and *EPCAM* exons, using Salsa MLPA P003 MLH1/MSH2 probemix (MRC-Holland, Amsterdam, Netherlands) andP008 PMS2 for PMS2 (MRC-Holland). Patients with LS-associated class 4 (likely pathogenic) or class 5 (pathogenic) germline variants, based on the InSiGHT classification criteria, were diagnosed with LS.

LS diagnoses were not possible for deceased patients with isolated loss of MSH6 in the UUTC at the time of publication due to low quality urothelial tissue (normal) [20]. Thereafter, the genetic analysis of this proband, demonstrated the insertion of retrotransposon in the exon 5 of *MSH6*, which could be explained as a cause of LS [24]. Therefore, the patient was regarded as LS in the present study. Germline variants were categorized into class 3, according to the InSiGHT classification criteria, were regarded as variants of uncertain significance (VUS). Patients with VUS were not categorized into either LS or LLS.

#### LLS-associated tumors

Tumors without germline MMR pathogenic variants or somatic *MLH1* hypermethylation were classified as LLS-associated tumors. Somatic variants and copy number variations were investigated using Sanger sequencing (performed using DNA extracted from the micro dissected tissue specimens) and the MLPA method as described previously [12, 16–23].

### Whole exome sequencing for LLS cases

WES analysis was conducted at a private laboratory, Novogene Co., Ltd. via Chemical Dojin Co., Ltd., as follows: The Agilent SureSelect Human All Exon V6 (58 M) kit (Agilent Technologies Inc., Santa Clara, CA, USA) was used for DNA target enrichment, followed by sequencing with an Illumina HiSeq4000 sequencer (Illumina, Inc.) as described previously [25].

## Results

### Incidence of MMR-D tumors

**Fig. 1:**
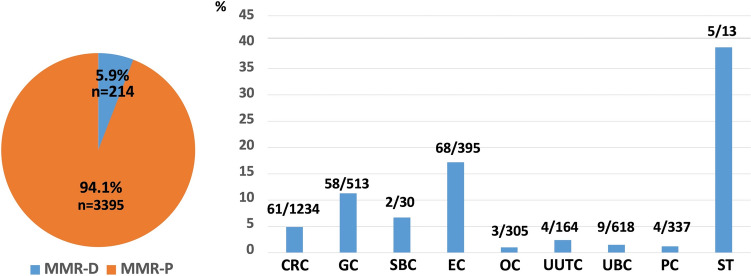
Percentage of patients with MMR-D tumor by different organs. *ST* sebaceous tumor, *OC* ovarian cancer, *CRC* colorectal cancer, *EC* endometrial cancer, *SBC* small-bowel cancer, *GC* gastric cancer, *PC* prostatic cancer, *UUTC* upper urinary tract cancer, *UBC* urinary bladder cancer, *MMR* mismatch repair, *MMR-D* loss of expression of at least one MMR protein, *MMR-P* expression of all MMR proteins

The total MMR-D incidence among the study population was 5.9% (24/3609). The MMR-D incidence in descending order were as follows: ST (38.5%, 5/13), EC (17.2%, 68/395), GC (11.3, 58/513), SBC (6.7%, 2/30), CRC (4.9%, 61/1234), UUTC (2.4%, 4/164), UBC (1.5%, 9/618), PC (1.2%, 4/337), and OC (1.0%, 3/305) (Fig. 1).

### Pattern of MMR-D by MMR-IHC

**Fig. 2:**
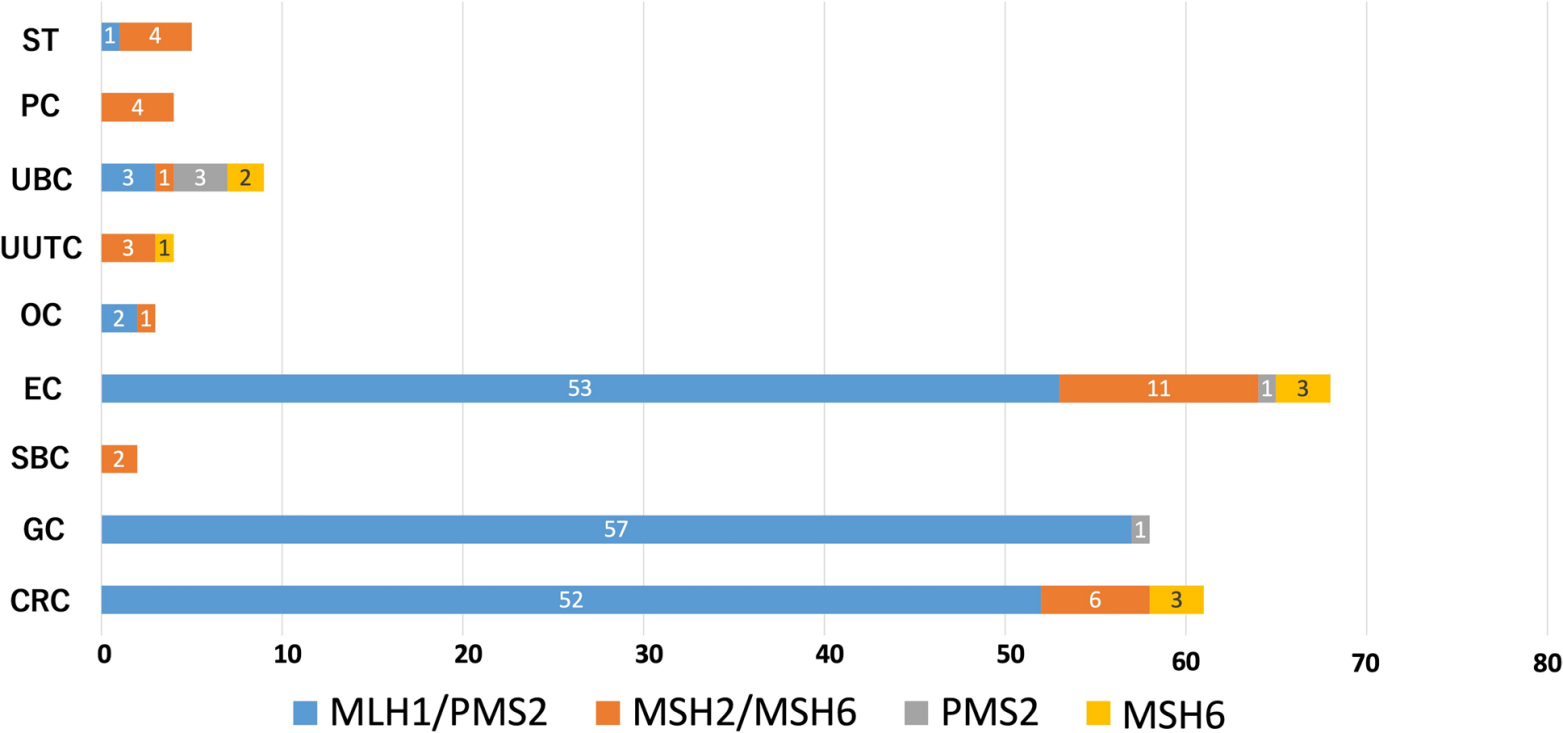
Number and MMR-D pattern by cancer type. *ST* sebaceous tumor, *OC* ovarian cancer, *CRC* colorectal cancer, *EC* endometrial cancer, *SBC* small-bowel cancer, *GC* gastric cancer, *PC* prostatic cancer, *UUTC* upper urothelial cancer, *UBC* urinary bladder cancer, *MMR-D* loss of expression of at least one MMR protein

Figure 2 demonstrated the pattern of MMR-D, identified using MMR-IHC. Upon analyzing tumors collectively, the identified MMR-D pattern was predominantly loss of expression of the MSH2/MSH6 dimer (90.0%, 9/10) followed by isolated loss of MSH6 (10.0%, 1/10) in UUTC, PC, and SBC. In contrast, in GC, CRC, and EC, the MMR-D pattern was predominantly MLH1/PMS2 (86.6%, 162/187). The MMR-D pattern in UBC varied, demonstrating loss of MLH1/PMS2 (n = 3), MSH2/MSH6 (n = 1), isolated loss of MSH6 (n = 3), and isolated loss of PMS2 (n = 2).

### MLH1-methylated tumors

**Fig. 3:**
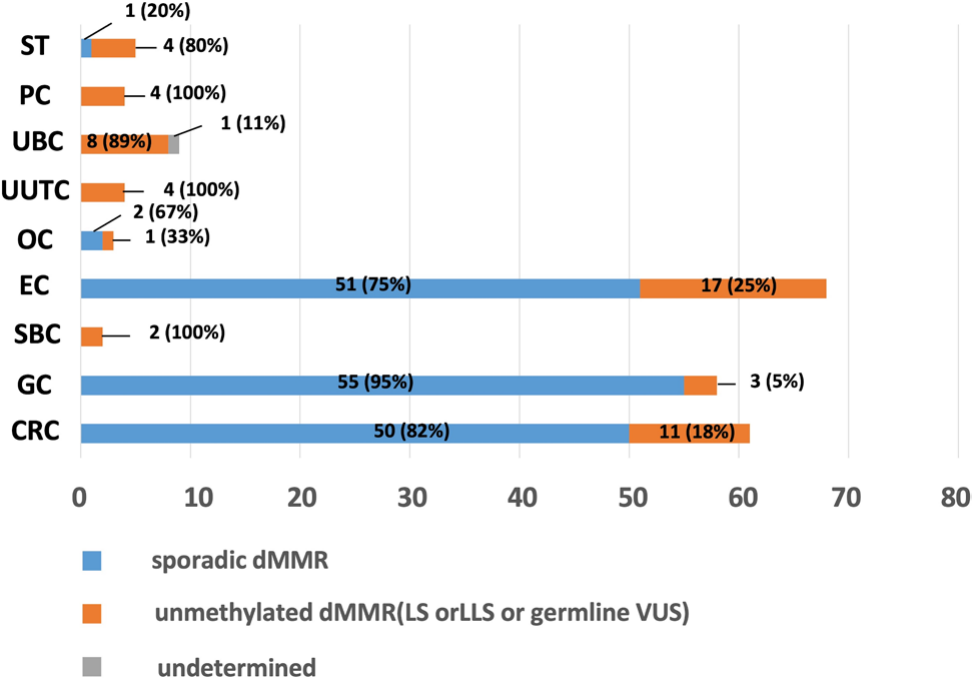
Number of cases by subtypes of MMR-D LS-related tumors. *ST* sebaceous tumor, *OC* ovarian cancer, *CRC* colorectal cancer, *EC* endometrial cancer, *SBC* small-bowel cancer, *GC* gastric cancer, *PC* prostatic cancer, *UUTC* upper urothelial cancer, *UBC* Urinary bladder cancer, *dMMR* deficient DNA mismatch repair, *LS* Lynch syndrome, *LLS* Lynch-like syndrome, *VUS* variants of uncertain significance

It can be seen in Fig. 3 that *MLH1*-hypermethylated tumors among MMR-D tumors were common in GC (94.8%, 55/58), CRC (82.0%, 50/61), and EC (75.0%,51/68). In contrast, *MLH1*-unmethylated tumors were common in UUTC (100%, 4/4), PC (100%, 4/4), SBC (100%, 2/2), and UBC (88.9%, 8/9).

### *MLH1*-unmethylated MMR-D tumors according to genetic testing

**Fig. 4:**
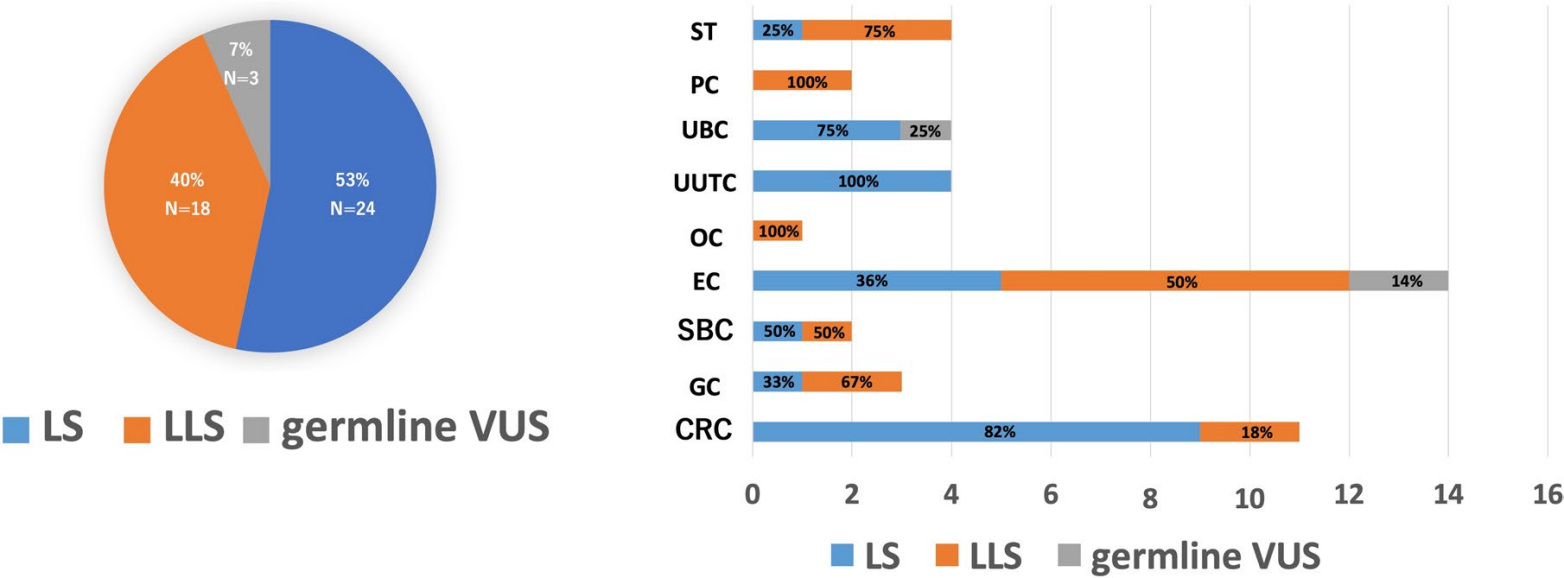
Number of cases by classification of LS, LLS and germline VUS after genetic testing for Lynch syndrome. *ST* sebaceous tumor, *OC* ovarian cancer, *CRC* colorectal cancer, *EC* endometrial cancer, *SBC* small-bowel cancer, *GC* gastric cancer, *PC* prostatic cancer, *UUTC* upper urothelial cancer, *UBC* urinary bladder cancer, *LS* Lynch syndrome, *LLS* Lynch-like syndrome, *VUS* variants of uncertain significance

Genetic testing was conducted for 47 out of the 58 patients with *MLH1*-unmethylated dMMR tumors. The patients were divided into three categories: LS (n = 26, 55%), LLS (n = 18, 38%), and germline VUS (n = 3, 7%) (Fig. 4). Germline VUS was exclusively identified in EC (n = 2) and UBC (n = 1). The incidence of LS among the *MLH1*-unmethylated patients was highest in the UUTC (100%, 4/4), followed by CRC (82%, 9/11), and UBC (75%, 3/4). Meanwhile, the incidence of LLS was highest in PC (100%, 4/4) and OC (100%, 1/1), followed by ST (75%, 3/4).

### Summary of MMR-D tumors based on molecular characteristics

Table 1 summarizes the molecular characteristics of the MMR-D tumors. That is, proportion of MMR-D, pattern of loss of MMR protein expression, proportion of *MLH1*-methylated tumors among all MMR-D tumors, proportion of *MLH1*-unmethylated tumors among all MMR-D tumors, proportion of LS and LLS among all *MLH1*-unmthylated tumors. The proportion of germline VUS is shown for EC and UBC.

**Table 1:** Summary of molecular characteristics in MMR-D tumors

Type of tumor	Pattern of MMR-D					*MLH1*-methylated MMR-D among all MMR-D cases	*MLH1*-unmethylated MMR-D among all MMR-D cases	Type of MLH1-unmethylated MMR-D			
	MMR-D (%)	Loss of MLH1/PMS2 among all MMR-D cases	Loss of MSH2/MSH6 among all MMR-D cases	Loss of MSH6 among all MMR-D cases	Loss of PMS2 among all MMR-D cases			LS (A%, B%, C%)	LLS (A%, B%, C%)	Germline VUS (A%, B%, C%)	N.A (genetic testing not performed) (A%, B%, C%)
CRC	4.9 (61/1234)	52 (85.2)	6 (9.8)	3 (4.9)	0 (0)	50 (82.0)	11 (18.0)	9 (81.8%, 14.8%, 0.7%)	2 (18.2%, 3.3%, 0.2%)	0	0
GC	11.3 (58/513)	57 (98.3)^a^	0 (0)	0 (0)	1 (1.7)	55 (94.8)	3 (5.2)	1 (33.3%, 1.7%, 0.2%)	2 (66.7%, 3.4%, 0.4%)	0	0
SBC	6.7 (2/30)	0 (0)	2 (100)	0 (0)	0 (0)	0 (0)	2 (100)	1 (50.0%, 50.0%, 2.9%)	1 (50.0%, 50.0%, 2.9%)	0	0
EC	17.2 (68/395)	53 (77.9)	11 (16.2)^b^	3 (4.4)	1(1.5)	51 (75.0)	17 (25.0)	5 (29.4%, 7.4%, 1.3%)	7 (41.2%, 10.3%, 1.8%)	2 (11.8%, 2.9%, 0.5%)	3 (17.6%, 4.4%, 0.8%)
OC	1.0 (3/305)	2 (66.7)	1 (33.3)	0 (0)	0 (0)	2 (66.7)	1 (33.3)	0	1 (100%, 33.3%, 0.3%)	0	0
UUTC	2.4 (4/164)	0 (0)	3 (75.0)	1 (25.0)	0 (0)	0 (0)	4 (100)	4 (100%, 100%, 2.4%)	0	0	0
UBC	1.5 (9/618)	3 (33.3)	1 (11.1)	2 (22.2)	3 (33.3)	0 (0)	8 (88.9)	3 (37.5%, 33.3%, 0.5%)	0	1 (12.5%, 11.1%, 0.2%)	4 (50.0%, 44.4%, 0.6%)
PC	1.2 (4/337)	0 (0)	4 (100)	0 (0)	0 (0)	0 (0)	4 (100)	0	2 (50.0%, 50.0%, 0.6%)	0	2 (50.0%, 50.0%, 0.6%)
ST	38.5 (5/13)	1 (20.0)	4 (80.0)	0 (0)	0 (0)	1 (20.0)	4 (80.0)	1 (25.0%, 20.0,% 7.7%)	3 (75.0%, 60.0%, 23.1%)	0	0
Total	5.9 (214/3395)										

A%: Proportion among *MLH1*-unmethylated cases

B%:Proportion among MMR-D cases

C%: Proportion among all cases

*ST* sebaceous tumor, *OC* ovarian cancer, *CRC* colorectal cancer, *EC* endometrial cancer, *SBC* small-bowel cancer, *GC* gastric cancer, *PC* prostatic cancer, *UUTC* upper urinary tract cancer, *UBC* urinary bladder cancer, *MMR-D* loss of expression of at least one MMR protein

^a^Loss of MLH1/PMS2/MSH6 was categorized as loss of MLH1/PMS2

^b^Loss of isolated MSH2 were categorized as loss of MSH2/MSH6

### Characteristics of LLS and WES

**Fig. 5:**
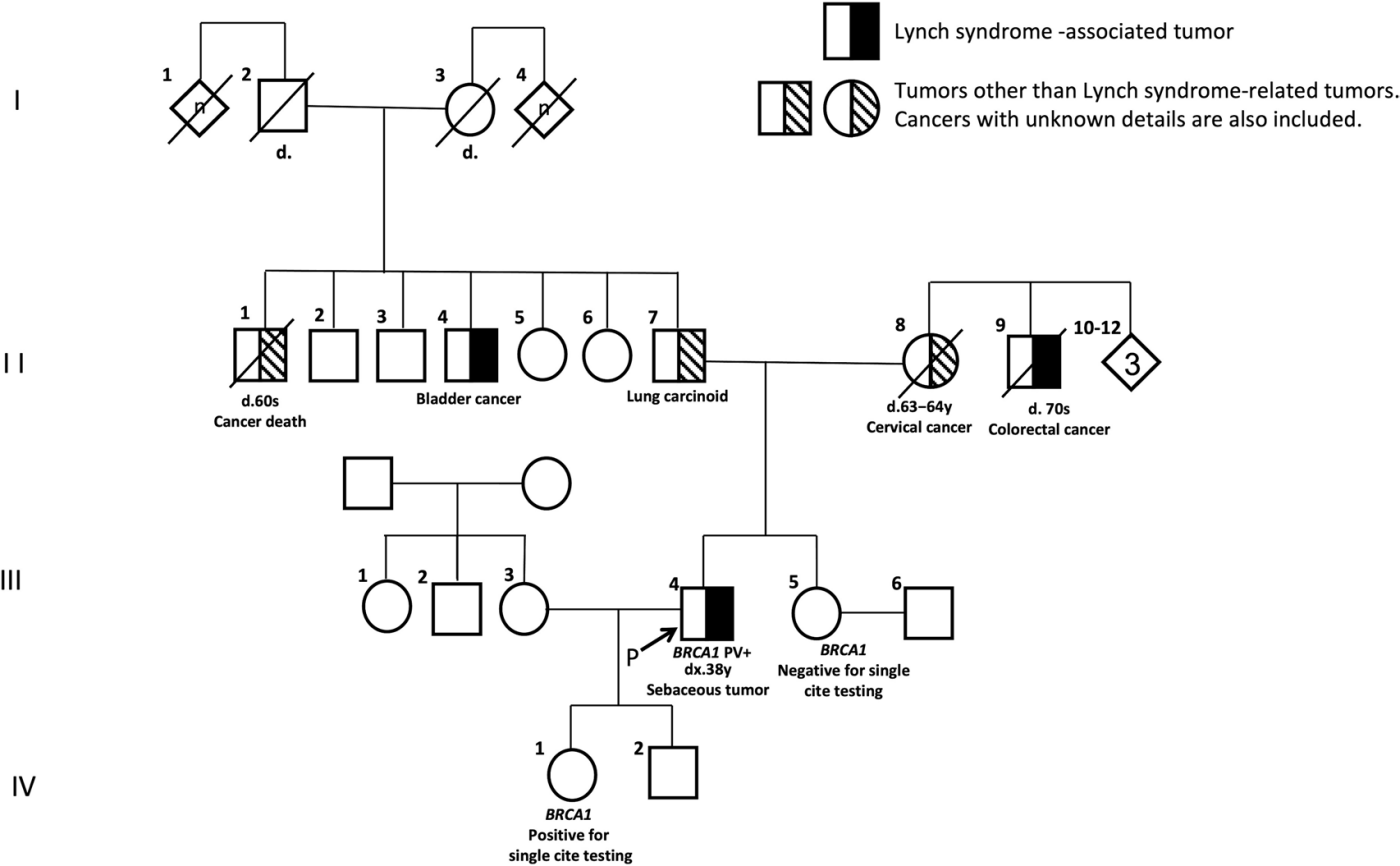
The pedigree of case 3. *PV* pathogenic variant

**Fig. 6:**
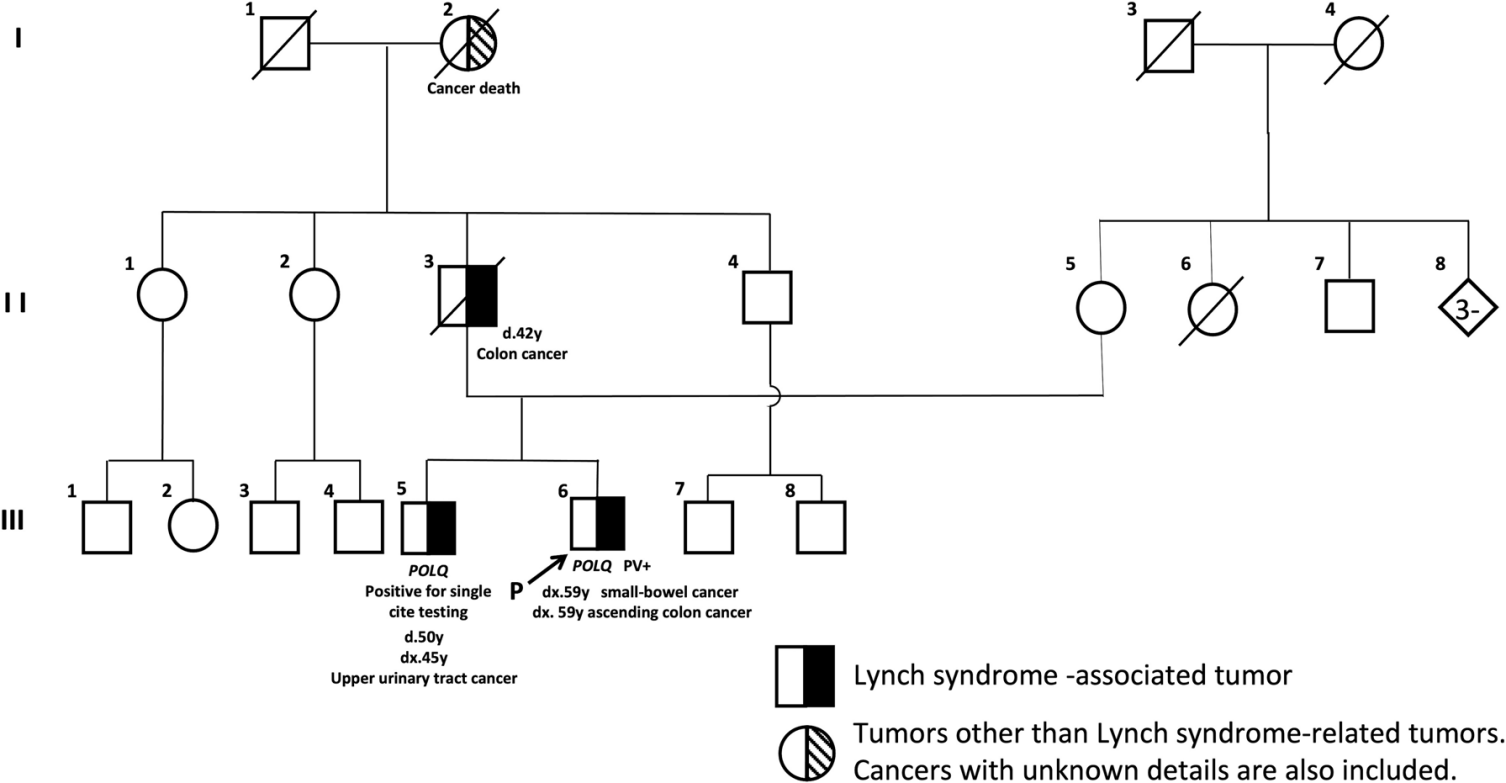
The pedigree of case 14. *PV* pathogenic variant

The characteristics of 18 patients with LLS are demonstrated in Table 2. Age at diagnosis of the index tumor ranged from 42 to 82 years (median, 66 years) and the male to female ratio was 8:10. CRC was confirmed, from medical history, in two patients with LS-associated tumors. No patients fulfilled the revised Amsterdam criteria [26]. Somatically inactivated MMR variants were successfully conducted in 16 patient samples. In 15 samples, one to two Class 4/Class 5 MMR gene variants were detected. In the remaining sample, two Class 3 variant was detected. Whole exome sequence was performed for 12 patient samples. Two patients demonstrated non-*MMR* germline pathogenic variants. A 63-year-old man (Case 3, III-4) (Fig. 5) with sebaceous cancer (MSH2/MSH6) was found to carry a *BRCA1*(NM_007294.4:c.2800C>T) pathogenic nonsense variant (supplementary Fig. 1), and a 57-year-old man (Case 14, III-6) (Fig. 6) with small-bowel cancer (MSH2/MSH6 loss) was found to carry a *POLQ* variant (NM_199420: c.245_250delinsTGTA) which probably affects function (supplementary Fig. 2). Multiple sebaceous tumors (MSH2/MSH6 loss) were identified in Case 3 and genetic analysis was performed, as previously reported [23], and an additional sebaceous tumor developed at the age of 57. Case 14: The older male sibling (III-5, Fig. 6) underwent respective surgery for UUTC at the age of 45 and died of the disease at the age of 50. The father of the siblings (II-3, Fig. 6) suffered a CRC-related mortality at the age of 42. The UUTC of the III-5 demonstrated loss of MSH2/MSH6 (supplementary Fig. 3). In addition, Sanger sequencing detected the *POLQ* variant, as identified in Case 14 (supplementary Fig. 4).

**Table 2:**
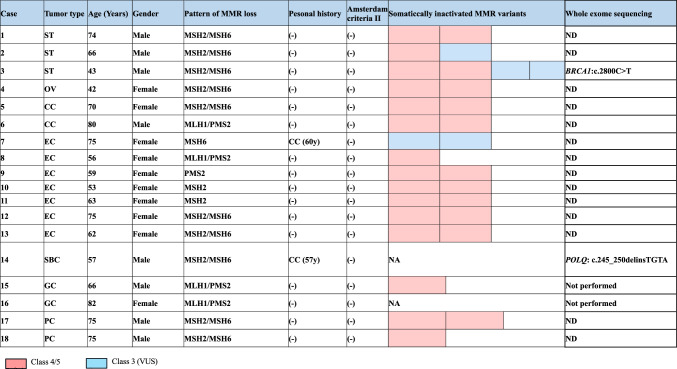
Summary of patients with LLS

*ST* sebaceous tumor, *OV* ovarian cancer, *CC* colon cancer, *EC* endometrial cancer, *SBS* small-bowel cancer, *GC* gastric cancer, *PC* prostatic cancer, *LLS* Lynch-like syndrome

## Discussion

We provided data on the incidence of dMMR tumors, assessed by MMR-IHC, among resected specimens of nine different tumor types. CRC, GC, SBC, EC, OC, UUTC, and ST are known to be LS-associated tumors, as listed in the revised Bethesda guidelines [27]. It remains controversial whether PC and UBC are associated with LS; however, and increased risk of PC and UBC in individuals with *MSH2* germline pathogenic variants has been reported [28, 29]. MMR-IHC allows for the identification of genes related to the alteration in dMMR function. Therefore, using MMR-IHC and *MLH1*-methylation analysis, we efficiently selected patients with dMMR tumors who were candidates for LS genetic testing. It is useful to determine the ratio of LS to LLS among LS suspected patients before genetic testing is performed.

To the best of our knowledge, this is the first comprehensive study that demonstrated the proportion of molecular-genetics-based categorized dMMR tumors across various tumors. The ratio of LS to LLS was 4:3. This suggests that approximately 50% of patients with a *MLH1*-unmethylated dMMR tumor would not have LS. Studies, with a specific focus on CRC and /or EC, demonstrated an LS to LLS ratio at 9:30 [9, 30]. Rodrigez et al. [9] analyzed 1705 CRC cases and reported an LS to LLs of 16:43. This ratio is lower than that (9:2) reported by Chika et al. [12]. It should be noted that the ratio can be influenced by the method and quality of LS genetic testing.

The term “LLS” is used for patients with dMMR tumors (specifically CRC and EC) with no *MLH1*, *MSH2*, *MSH6*, *PMS2*, or *EPCAM* deletion variants or *MLH1* somatic methylation. At least three molecular genetic possibilities have been considered to explain LLS-AT: cryptic/undetected germline pathogenic variants such as non-cording regions, inversions, or translocations of MMR genes that are not routinely detectable by current genetic testing, (occult/undetected LS) [31]; biallelic somatic variants in MMR genes of unknown etiology [32]; and germline pathogenic variants in non-MMR genes (heritable predisposition) [33]. The latter two may overlap in some patients.

The underlying germline variants of genes involved in LLS have been poorly explored. Some LLS-based studies [34, 35] reported the presence of biallelic germline variants in *MUTYH*. The *MUTYH*-associated polyposis can overlap with the LS phenotype, by somatic inactivation of MMR genes [34]. LLS patients carrying *POLE* and *POLD1* germline variants have also been identified [36, 37]. Whole exome sequencing identified germline pathogenic/likely pathogenic variants in DNA repair genes, such as *MCM8*, MCM9, *WRN*, *MCPH1, BARD1*, *REV3L*, *EXO1, POLD1*, *RFC1*, *RPA1, ATM,* and *MLH3*. In addition, other cancer-related genes, such as *PPARG*, *CTC1*, *DCC* and *ALPK,* were identified as candidate genes for LLS [13, 37–39].

In the present study, Case 3 carried a *BRCA1* (c.2800C>T; p.Q934X) pathogenic variant, which was reported as a founder mutation in the Japanese population [40]. It is well-known that *BRCA1/2* is involved in homologous recombination (HR), which is an error-free repair mechanism for DNA double-strand breaks (DSBs) [31]. Colorectal cancer (CRC) with HR deficiency (HRD), developed via biallelic somatic variants of HR-related genes, including *BRCA1/2*, were more frequent in MSI-H/dMMR than in MMS/pMMR, suggesting a significant association between alterations in the HRD pathway and dMMR [41, 42]. Although *BRCA1/2* variants might affect MSI status, there is no evidence to the support that *BRCA1/2* germline variants induce *MMR* somatic variants. It is possible that HRD cells rely on more error-prone alternative DNA damage response pathways, such as nonhomologous end joining, to repair DNA breaks and avoid mitotic catastrophe and cell death. Thus, increasing mutagenesis and genomic instability, inactivating BRCA and other factors to induce MMR variants. A patient suffered from a ST with loss of MSH2/MSH6 proteins, caused by two somatic pathogenic mutations, that is, a nonsense variant (c.2038C>T/p.Arg680*) and a deletion of exons 14–15 in *MSH2* [23]. Subsequently, four primary STs were resected, but none with confirmed MSH2 [23]. A previous report [43] suggested that somatic inactivation of the fragile histidine triad (FHIT) gene associated with MSS or inactivation of the MMR system resulting in MSI contributes to the development of periocular sebaceous gland carcinomas in presumptive Muir-Torre syndrome. Moreover, Becker et al. [44] reported that somatic *BRCA1* deleterious mutations were associated with candidate tumor suppressor FHIT inactivation in sebaceous gland carcinomas with MSS. Therefore, there may be two possible mechanisms for ST pathogenesis. ST develops either on the background of a dMMR system with MSI via inactivating variants preferentially in *MSH2* or on the basis of HRD via deletions of *BRCA1* associated with MSS. Based on these pathways, the first tumor may have developed by the former mechanism and the remaining three tumors by the latter mechanism, though the FHIT inactivation was not examined.

We identified the heterozygous *POLQ* germline frameshift variant in a LLS patient (Case 14). *POLQ* is involved in the repair of DSBs. Most DSBs are repaired by two pathways: one is the canonical nonhomologous end joining (c-NHEJ), which directly relegates DNA ends without extensive processing; the other is the HR pathway, which is the only precise DSB repair pathway. *POLQ* was found to be s involved in a third pathway termed polymerase theta-mediated end joining (TMEJ) [45]. Whether POLQ suppresses or promotes genomic instability still remains unknown. TMEJ repair is error prone and is known to generate genomic translocations. Raskin et al. [46] reported that *POLQ* germline pathogenic variants were detected in each LLS patient and sporadic colorectal cancer patient with MSS. Belhadj et al. [47] identified seven *POLQ* germline pathogenic variants in patients with suspected LS along with diagnosis based on AC I/II (n = 58) and BG (n = 385) criteria. Since the *POLQ* variants were not found to be significantly enriched in the study population, lack of association with CRC predisposition was suggested. *POLQ* has multiple functions, which varies based on the coordinating proteins. This suggests that *POLQ* may be one of the causative factors for LLS; however, it may not be directly related to the development of LLS.

The limited data on germline alterations in patients with LLS suggest that hereditary factors should not be excluded; however, further investigations are required. In addition, patients suspected to have LLS should be offered genetic counseling that discusses updated germline variant data, which includes non-*MMR* genes.

The present study has several limitations. First, the data was obtained from a single institution, analyzed retrospectively with a small sample size for each tumor type. Second, data of clinicopathological and personal/family histories regarding the development of malignant neoplasms could not be compared between patients with LS and LLS, especially due to the lack of data in patients with LLS. Furthermore, LS-associated tumors, such as brain, bile duct, and pancreatic tumors could not be analyzed.

Nevertheless, this is the first report that comprehensively documents the characteristics of various dMMR tumors. Notably, our data regarding the LS to LLS ratio would be useful for genetic counseling in patients who are suspected to have LS. Genetic mechanisms for the pathogenesis of LLS needs further investigation.

### Supplementary Information

Below is the link to the electronic supplementary material.

Supplementary file1 (DOCX 16 KB)

Supplementary file2 Supplementary Figure 1 Confirmation of BRCA1:c.2800C>T (p.Gln934*) in case 3 (JPG 1686 KB)

Supplementary file3 Supplementary Figure 2 Confirmation of POLQ: c.245_250delinsTGTA (p.Glu82Valfs*2) in case 14 (JPG 1699 KB)

Supplementary file4 Supplementary Figure 3 Immunohistochemistry for MMR protein in UUTC of the III-5. Loss of expression of MSH2 and MSH6 proteins was observed (JPG 5336 KB)

Supplementary file5 Supplementary Figure 4 Confirmation of POLQ: c.245_250delinsTGTA (p.Glu82Valfs*2) in the III-5 (JPG 1119 KB)

## Data Availability

The datasets during and/or analyzed during the current study available from the corresponding author on reasonable request.
