# Supplementary material for: Incidence and molecular characteristics of deficient mismatch repair conditions across nine different tumors and identification of germline variants involved in Lynch-like syndrome
Source: Int J Clin Oncol. 2024 Apr 14;29(7):953–63. doi: 10.1007/s10147-024-02518-y (PMC11196295; doi:10.1007/s10147-024-02518-y)
Supplement: Supplementary file 1 — Supplementary file1 (DOCX 16 KB) [file 10147_2024_2518_MOESM1_ESM.docx]

supplementary Table 1

Reports of dMMR, LS, and LLS identified by immunohistochemistry

| **Tumor type** | **Case accumulation period** | **Number of Patients** | **Male / Female** | **Age（years）^a^** | **Publication** |
| --- | --- | --- | --- | --- | --- |
| **Colorectal cancer** | **March 2005 - April 2014** | **1234** | **752/482** | **69 (24-97)** | **Jpn J Clin Oncol. 2017;47:108-117.** |
| **Gastric Cancer** | **April 2005- June 2016** | **513** | **378/135** | **70 (22-99)** | **Jpn J Clin Oncol. 2021;51:886-894.** |
| **Small-bowel cancer** | **March 2002- March 2017** | **30** | **16/14** | **64 (17-87)** | **J Anus Rectum Colon. 2020;4(4):165-173.** |
| **Endometrial cancer** | **January 2005- December 2015** | **395** | **0/395** | **59 (26-87)** | **Jpn J Clin Oncol. 2021;51:60-69.** |
| **Ovarian cancer** | **April 2005 - September 2014** | **305** | **0/305** | **54 (18-83)** | **Jpn J Clin Oncol. 2018;48:728-735.** |
| **Upper urinary tract cancer** | **March 2005- November 2017** | **164** | **124/40** | **72 ( 39-88)** | **Jpn J Clin Oncol. 2020;50:80-88.** |
| **Urinary bladder cancer** | **June 1997- February 2018** | **618** | **494/124** | **70 (24-97)** | **Int J Clin Oncol. 2021;26:1524-1532.** |
| **Prostatic cancer** | **2001年1月- 2016年5月** | **337** | **337/0** | **67 (52-81)** | **Jpn J Clin Oncol. 2021;51(4):639-645.** |
| **Sebaceous tumor** | **2005年1月- 2014年3月** | **13** | **7/6** | **70.5 (43-90)** | **Jpn J Clin Oncol. 2018;48:514-521.** |
| **Total** |  | **3609** |  |  |  |

a : Median (range)

dMMR: deficient DNA mismatch repair, LS: Lynch syndrome, LLS: Lynch-like syndrome
