# Supplementary figures and images for: Incidence and molecular characteristics of deficient mismatch repair conditions across nine different tumors and identification of germline variants involved in Lynch-like syndrome
Source: Int J Clin Oncol. 2024 Apr 14;29(7):953–63. doi: 10.1007/s10147-024-02518-y (PMC11196295; doi:10.1007/s10147-024-02518-y)

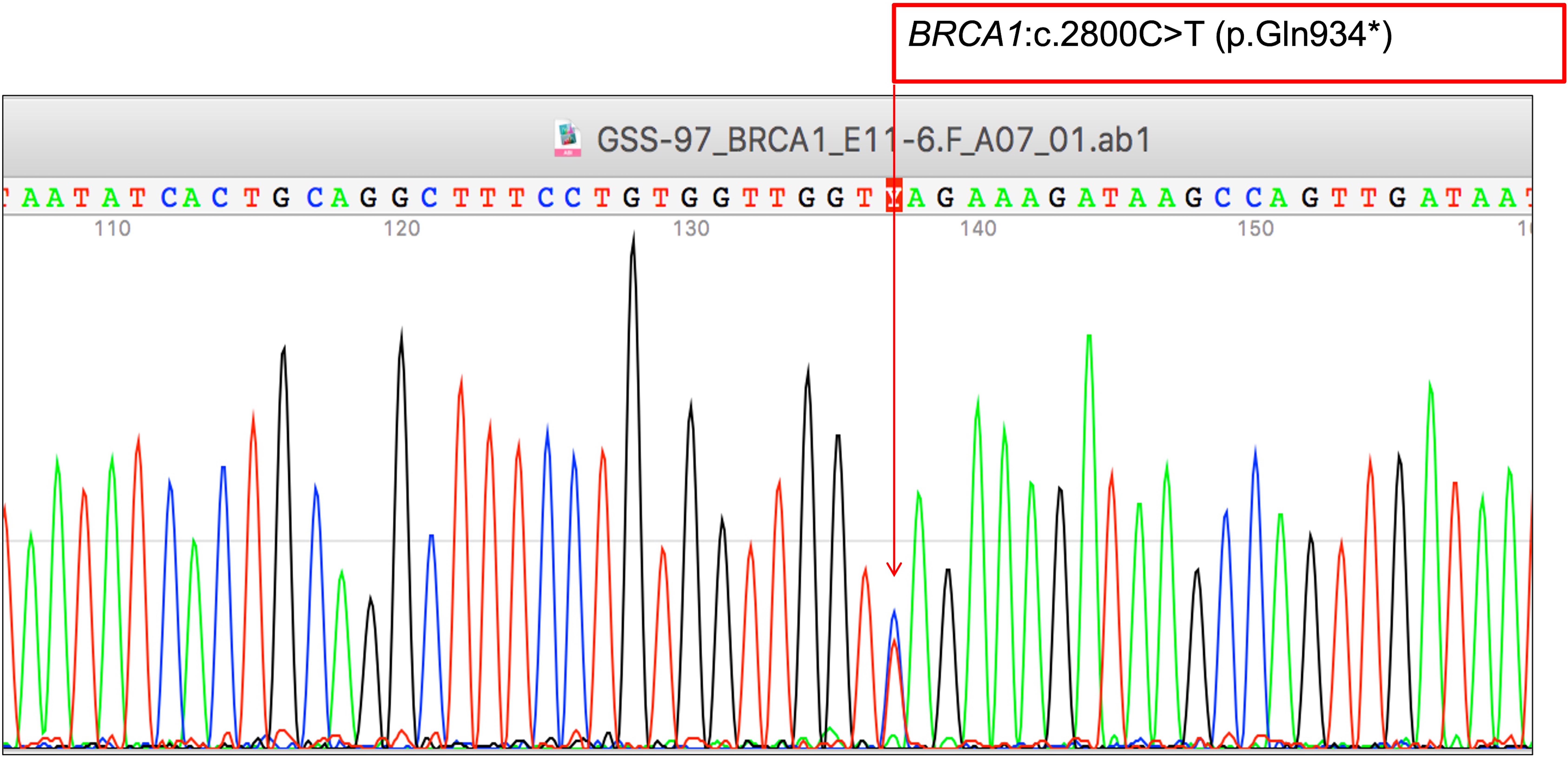

Supplement: Supplementary file 2 — Supplementary file2 Supplementary Figure 1 Confirmation of BRCA1:c.2800C>T (p.Gln934*) in case 3 (JPG 1686 KB) [file 10147_2024_2518_MOESM2_ESM.jpg]

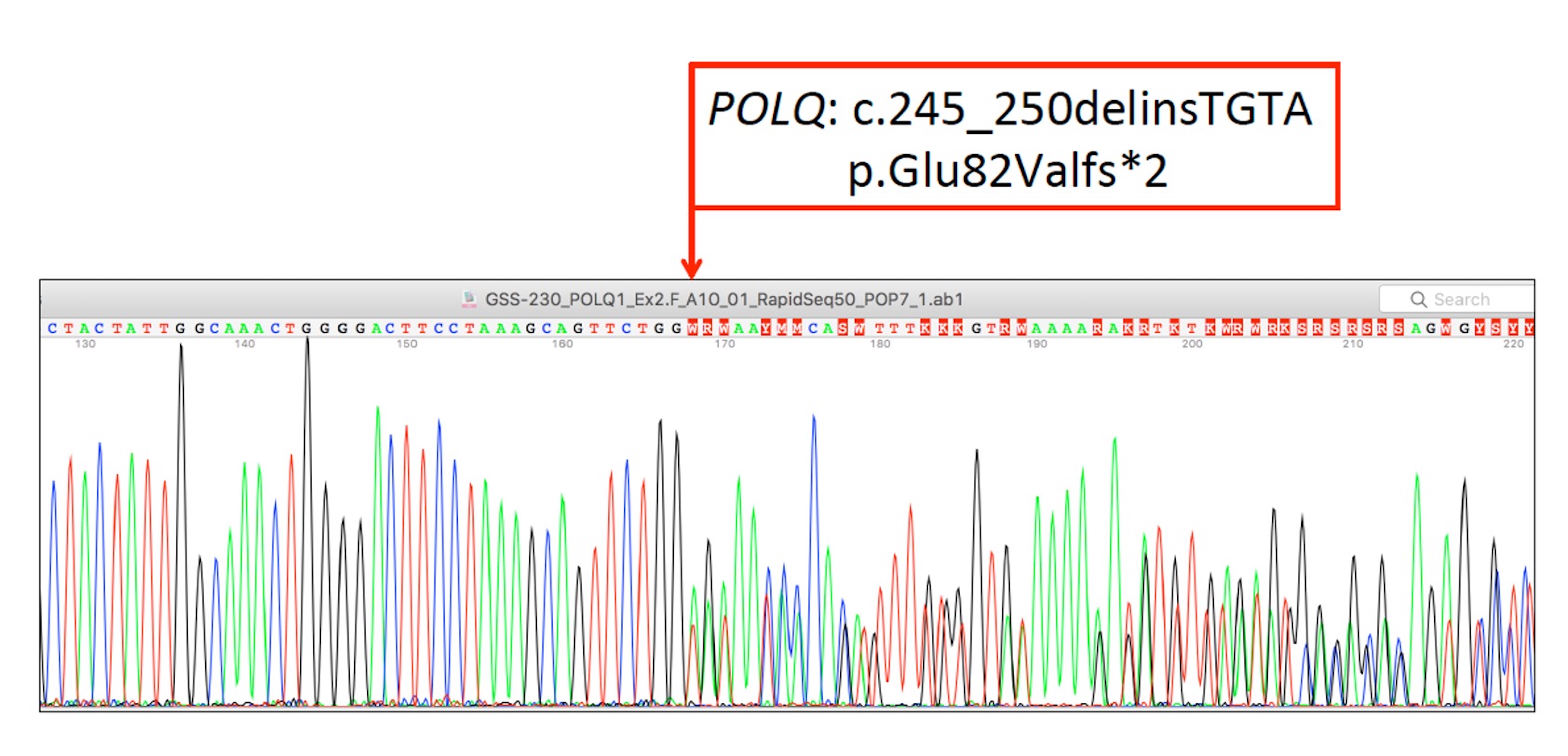

Supplement: Supplementary file 3 — Supplementary file3 Supplementary Figure 2 Confirmation of POLQ: c.245_250delinsTGTA (p.Glu82Valfs*2) in case 14 (JPG 1699 KB) [file 10147_2024_2518_MOESM3_ESM.jpg]

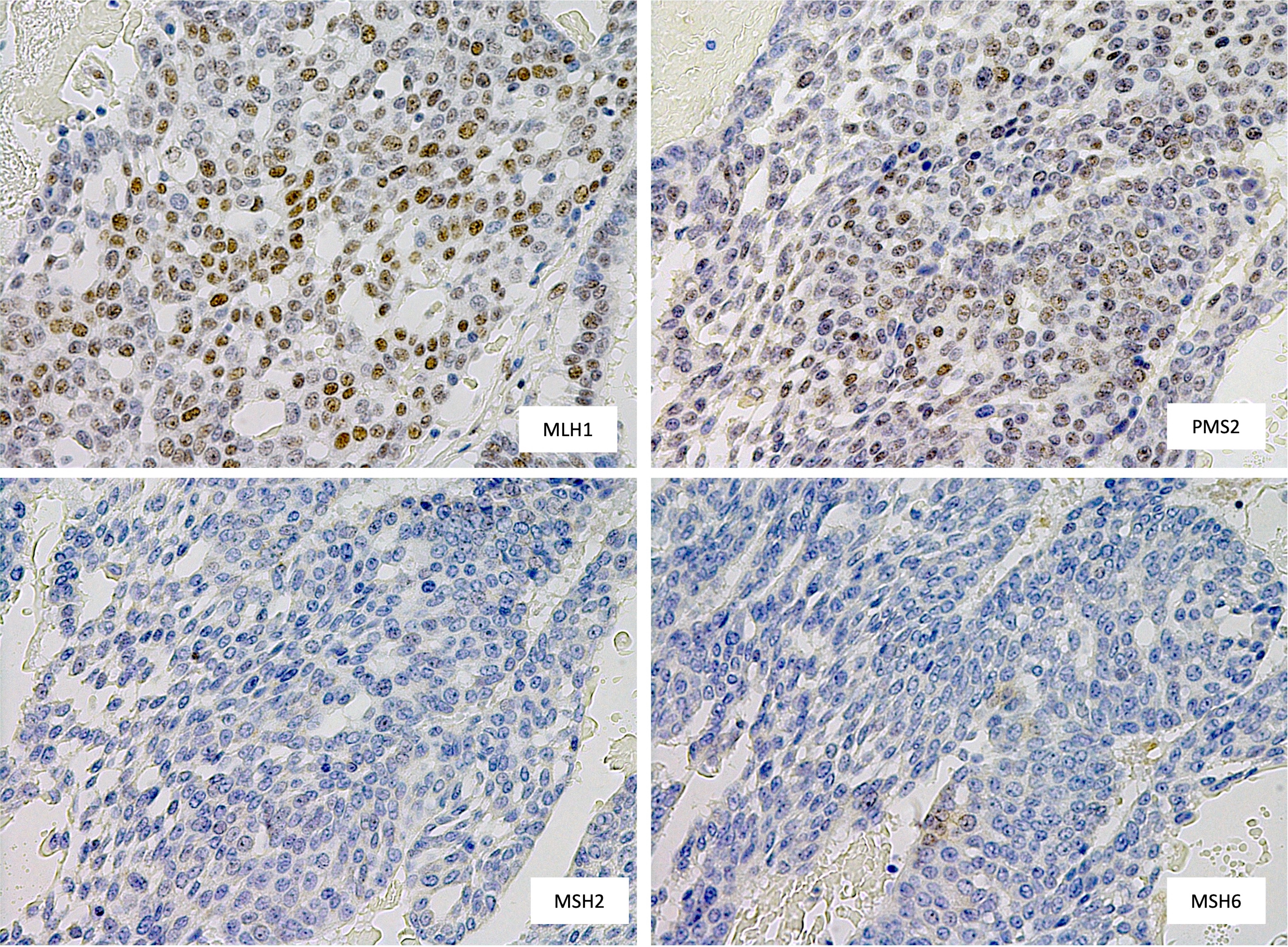

Supplement: Supplementary file 4 — Supplementary file4 Supplementary Figure 3 Immunohistochemistry for MMR protein in UUTC of the III-5. Loss of expression of MSH2 and MSH6 proteins was observed (JPG 5336 KB) [file 10147_2024_2518_MOESM4_ESM.jpg]

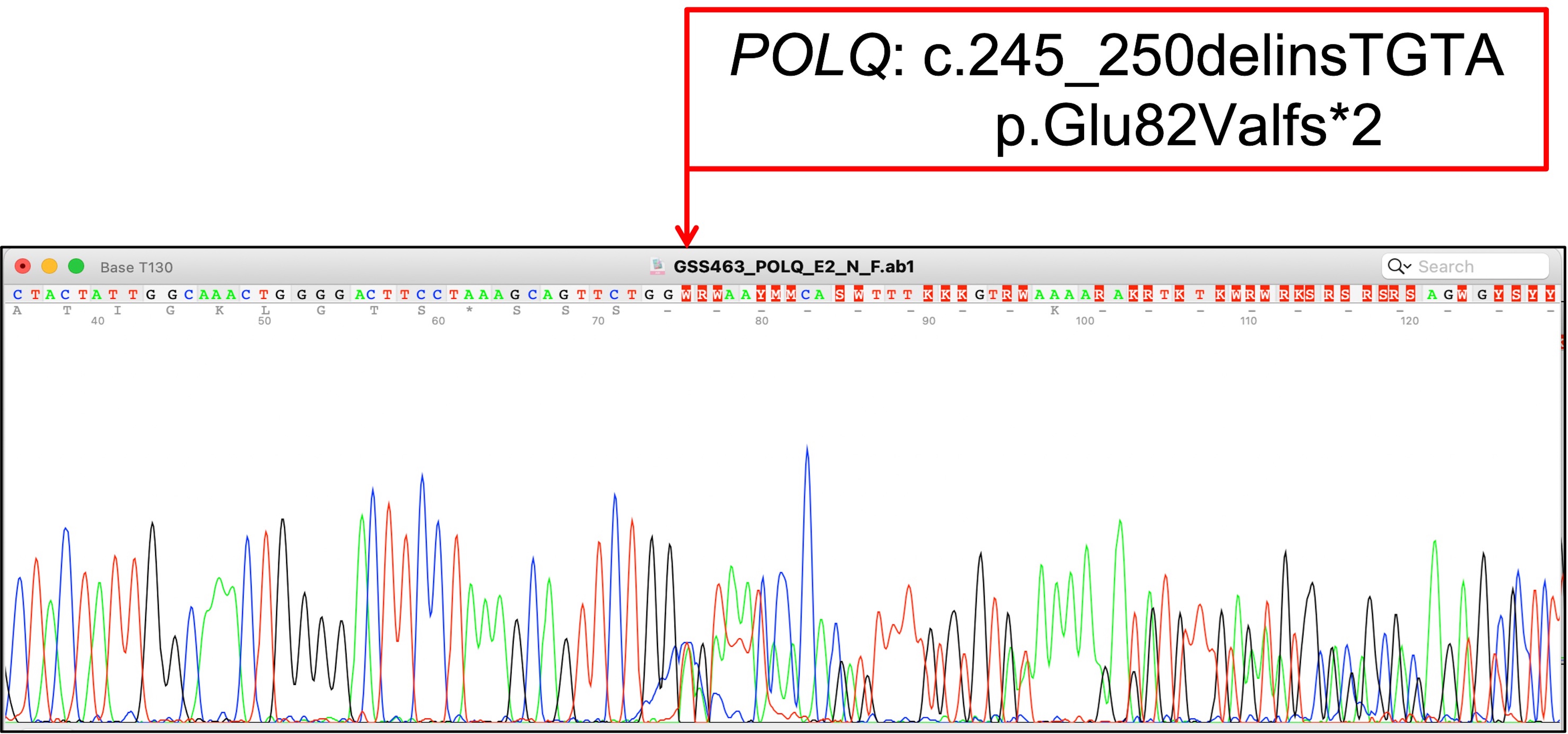

Supplement: Supplementary file 5 — Supplementary file5 Supplementary Figure 4 Confirmation of POLQ: c.245_250delinsTGTA (p.Glu82Valfs*2) in the III-5 (JPG 1119 KB) [file 10147_2024_2518_MOESM5_ESM.jpg]
